# Thioredoxin in Post-Injury Sepsis And Ards

**DOI:** 10.1186/2197-425X-3-S1-A34

**Published:** 2015-10-01

**Authors:** J Eriksson, A Gidlöf, O Brattström, B Persson, E Larsson, A Oldner

**Affiliations:** Karolinska Institutet, Physiology and Pharmacology, Section of Anesthesiology and Intensive Care, Stockholm, Sweden; Karolinska University Hospital, Solna, Anesthesiology, Surgical Services and Intensive Care, Stockholm, Sweden

## Introduction

Trauma is one of the leading causes of mortality worldwide, resulting in a great global burden of disability and mortality [1, 2]. Approximately half of all trauma deaths occur during the first hours due to traumatic brain injury or massive bleeding. Later deaths are due to, for example, sepsis or multiple organ failure [3]. Independent risk factors for post-injury sepsis are still somewhat poorly defined. Injury Severity Score (ISS), male gender, age, low Glasgow Coma Scale (GCS) and massive blood transfusion have been suggested as risk factors [4].

Thioredoxin (TRX) is thought to have important anti-oxidant properties but it also functions as an endogenous anti-inflammatory mediator [5]. The link between high plasma levels of TRX and sepsis has been studied previously, although with conflicting evidence [5, 6]. TRX-levels in trauma patients and the possible correlation to secondary complications such as post-injury sepsis and acute respiratory distress syndrome (ARDS) have to our knowledge not been studied previously.

## Objectives

To study the relationship between trauma and plasma levels of TRX as well as the possible correlation between post-traumatic plasma-TRX and secondary outcomes such as severe sepsis and ARDS.

## Methods

ICU-admitted trauma patients with an expected stay of >3 days (n = 84) were included. Median ISS was 29. Plasma-TRX was analyzed on day 1 and 3. Clinical, physiological and outcome data were retrieved from the trauma and ICU research registries. in addition, we analyzed plasma-TRX in 10 healthy subjects.

## Results

A three-fold increase in day 1 plasma-TRX was seen in trauma patients when compared to healthy volunteers (median, IQR 63.9 ng/ml, 39.3-114.6 vs. 22.6 ng/ml, 16.1-25.8 p = 0.0001). High ISS (>25) was associated with high plasma-TRX (median, IQR 72.1 ng/ml, 45.2-129.3 vs. 47.9 ng/ml, 35.0-81.2 p = 0.049).

TRX decreased significantly between day 1 and 3 (median, IQR 63.9 ng/ml, 39.3-114.6 vs. 38.6 ng/ml, 32.4-57.1 p < 0.0001).

There was no significant difference between survivors and non-survivors in plasma-TRX.

Day 1 plasma-TRX levels were significantly increased in patients who later developed severe sepsis compared to those who did not (median, IQR 72.9 ng/ml, 44.8-137.3 vs. 49.8 ng/ml, 39.0-78.3 p = 0.014), no such difference was noted for post-injury ARDS.

**Figure 1:**
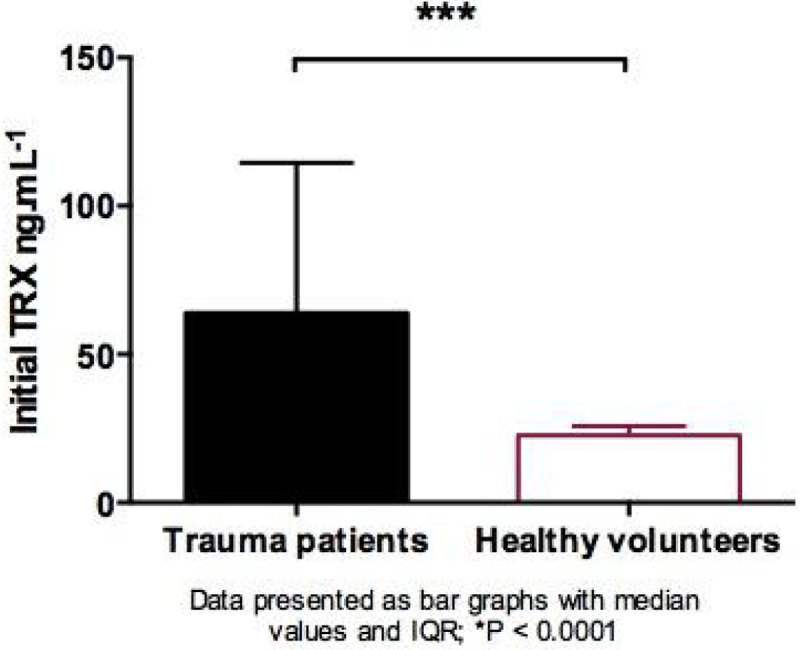
**Bar graph 1st TRX vs ctrl.**

**Figure 2:**
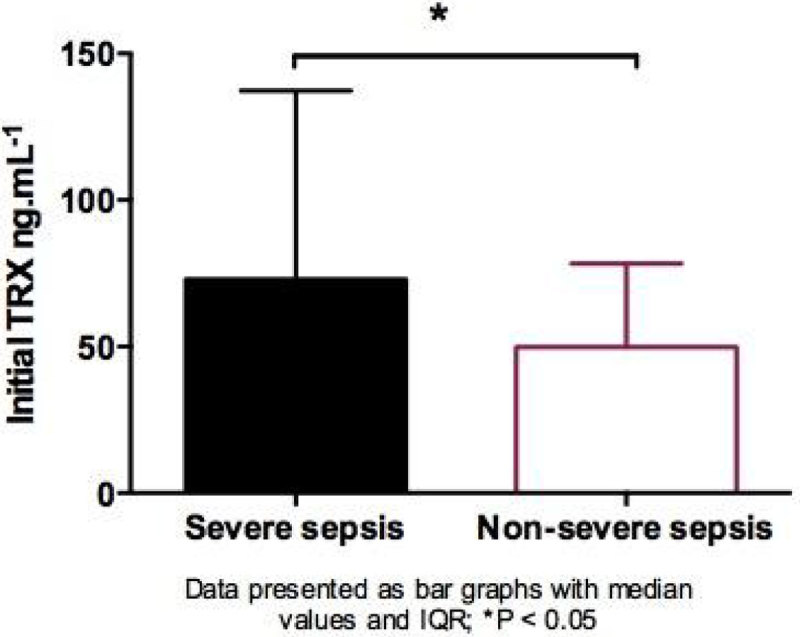
**Bar graph sev sepsis.**

## Conclusions

TRX levels rise after trauma. Increased plasma-TRX levels are associated with post-injury sepsis. The potential usefulness of TRX as a biomarker in trauma patients needs further evaluation in larger studies.

## Grant Acknowledgment

ALF-funding through Stockholm County Council and Karolinska Institutet.
